# Time to Change Our Viewpoints to Assess Renal Risks in Patients with Solitary Kidneys beyond Traditional Approaches?

**DOI:** 10.3390/jcm12216885

**Published:** 2023-10-31

**Authors:** Alper Alp, Ercan Saruhan, Emrah Doğan, Dilek Gibyeli Genek, Bülent Huddam

**Affiliations:** 1 Department of Nephrology, Faculty of Medicine, Mugla Sıtkı Koçman University, 48000 Mugla, Turkey; dilekgibyeli@mu.edu.tr (D.G.G.); bulenthuddam@mu.edu.tr (B.H.); 2 Department of Medical Biochemistry, Faculty of Medicine, Mugla Sıtkı Koçman University, 48000 Mugla, Turkey; ercansaruhan@mu.edu.tr; 3 Department of Radiology, Faculty of Medicine, Mugla Sıtkı Koçman University, 48000 Mugla, Turkey; emrahdogan@mu.edu.tr

**Keywords:** solitary functioning kidney, ambulatory blood pressure monitoring, soluble urokinase plasminogen activator receptor, procollagen type III N-terminal peptide, homocysteine

## Abstract

Solitary functioning kidney (SFK) can be defined as the absence or hypofunction of a kidney due to acquired or congenital reasons. A congenital solitary functioning kidney (cSFK) is more common than is an acquired one (aSFK) and is characterized by the anatomical absence (agenesis) or hypofunction (hypoplasia; hypodysplasia) of one kidney from birth. Among the acquired causes, the most important is nephrectomy (Nx) (due to the donor, trauma or mass resection). Patients with SFK are at risk for the development of chronic kidney disease (CKD) in the long term. This risk potential is also significantly affected by hypertension. The relationship between hypertension and subclinical chronic inflammation is a connection that has not yet been fully clarified pathogenetically, but there are many studies highlighting this association. In recent years, studies examining different fibrosis and inflammation biomarkers in terms of the evaluation and prediction of renal risks have become increasingly popular in the literature. Oxidative stress is known to play an important role in homocysteine-induced endothelial dysfunction and has been associated with hypertension. In our study, we aimed to investigate the relationship between ambulatory blood pressure monitoring (ABPM) and urinary/serum fibrosis and inflammatory markers in patients with SFK. We prospectively investigated the relationship between ABPM results and soluble urokinase plasminogen activator receptor (suPAR), procollagen type III N-terminal peptide (PIIINP), homocysteine and other variables in 85 patients with SFK and compared them between cSFK and aSFK groups. In the etiology of SFK, a congenital or acquired origin may differ in terms of the significance of biomarkers. In particular, the serum homocysteine level may be associated with different clinical outcomes in patients with cSFK and aSFK.

## 1. Introduction

Congenital anomalies of the kidney and urinary tract (CAKUT) are responsible for nearly 30% of all prenatal anomalies. The CAKUT general rate in live and stillbirths, respectively, is 0.3 to 1.6 per 1000 [1,2] and SFK constitutes an important segment within CAKUT. Unilateral renal agenesis (URA)/hypoplasia is the absence and inadequate development of unilateral renal parenchymal tissue resulting from disorders in the embryological metanephric development phase. Its frequency is approximately 0.04–0.05%. It constitutes 5% of all kidney malformations. Clinically, it may be accompanied by different renal and/or extrarenal anomalies.

In patients with SFK in an intact kidney, there can be a continuous increase in hyperfiltration, primarily to keep the glomerular filtration rate (GFR) at a stable level. Secondary to glomerular hypertension that develops over time, glomerular sclerosis develops, and a decrease in the number of healthy nephrons can be observed, which is an important risk factor for proteinuria and hypertension. The onset of compensatory renal hypertrophy, which has been described in patients with both cSFK and aSFK, is faster in patients with aSFK. With the reflex of remaining nephrons to provide a rapid stabilizing response to the sudden decrease in GFR, the glomerular workload increases and the progression to glomerulosclerosis and renal fibrosis accelerates.

Hypertension is the leading cause of cardiovascular disease and premature death. As a result of the increasingly aging world population, it is predicted that the prevalence of hypertension will reach 1.5 billion people globally in 2025 [3]. According to World Health Organization data, it is estimated that 1.28 billion people between the ages of 30 and 79 have hypertension. According to the same data, it is thought that approximately 46% of adults are unaware that they have hypertension, and only 21% of patients under treatment are at target values. Identifying/diagnosing asymptomatic patients, especially in patient groups at risk for CKD, is important.

ABPM has been accepted as the reference standard for the diagnosis of hypertension and is considered to be a better indicator of potential cardiovascular events compared to traditional office-based blood pressure measurements. Current guidelines state that ABPM should be preferable to office-based blood pressure measurements in diagnostic/screening examinations [4]. Studies conducted in recent years using this method, which provides the opportunity for evaluation over a 24 h period, with day and night periods evaluated separately, indicate that sleep blood pressure has a stronger relationship with clinical results than does awake blood pressure measured via ABPM [5].

In patients with SFK, serious clinical conditions such as proteinuria, hypertension and progression to CKD may occur in the long term, requiring periodic follow-up [6]. Controlling hypertension and proteinuria/albuminuria and slowing the progression to CKD are the most important goals in the treatment and follow-up approach. CKD is a progressive disease and a potent risk factor for cardiovascular diseases. Many urinary fibrosis markers have been used in clinical studies to predict CKD progression and show the level of renal fibrosis. One of these is type PIIINP. It is released into the urine during type III collagen deposition in the extracellular matrix. It has been shown in some studies to have a significant correlation with the level of tubulointerstitial fibrosis in renal biopsy series, and it has been suggested that it may be an independent predictor of a loss of renal function, especially in elderly patients [7].

suPAR is the systemic circulating (soluble) form of a membrane protein expressed by immune and endothelial cells, fibroblasts, cancer cells and vascular smooth muscle cells. suPAR serum levels reflect immune and inflammatory activation and are correlated with well-known inflammatory markers such as CRP and IL-6. It has been stated that it may play a role in the etiopathogenesis of renal diseases. It has been shown in a large cohort study that high levels may be associated with CKD progression [8].

The relationship between hypertension and serum homocysteine has been evaluated in many studies, although it is not clear from a pathogenetic perspective. Specifically, homocysteine can induce oxidative stress in endothelial cells by mediating the production of reactive oxygen species or by impairing the antioxidant system [9]. As a result, results such as vascular toxicity and hypertension occur.

Our hypothesis was that when we compare patients with SFK etiologically (congenital vs. acquired), markers such as ABPM, homocysteine, PIIINP and suPAR levels may differ in predicting renal risk factors. The fact that most studies conducted in this population have evaluated patients with SFK holistically in a heterogeneous manner and that the comparisons between congenital and acquired groups did not go beyond traditional risk factors led us to this study. The aim of our study was to review the hypertension control with ABPM measurements in patients with solitary kidneys who are routinely followed up in our center, to evaluate their renal functions and to evaluate the relationship of urinary/serum fibrosis and inflammatory markers with these parameters and other clinical and routine laboratory findings.

## 2. Materials and Methods

### 2.1. Study Design and Participants

This was a prospective, observational, cross-sectional study. Patients with SFK followed in nephrology outpatient clinics were included in the study, which was carried out between 1 June 2023 and 1 September 2023. After ethics committee approval was obtained, demographic, chronic disease and medication data of the volunteers who wanted to participate in the study and met the inclusion criteria were recorded. The diagnosis of solitary kidney was made using an abdominal ultrasonography + scintigraphy and/or abdominal computed tomography protocol. Routine biochemical examinations, hemogram and hemogram sub-parameters, 24 h urine protein, albumin, sodium, and potassium excretion, and PIIINP and suPAR were also measured in the same 24 h urine samples. Urine samples in Eppendorf tubes were stored at −80 °C until the period of study. ABPM measurements were made. Laboratory findings and their relationships with demographic data and ambulatory blood pressure results were investigated.

### 2.2. Inclusion and Exclusion Criteria

The study population consisted of patients between the ages of ≥18 and <80, who were followed up in the adult nephrology outpatient clinic, had URA/hypoplasia and unilateral Nx, and did not receive renal replacement therapy. Patients with active solid organ and hematological malignancy (renal cell carcinoma patients who were metastatic or receiving chemotherapy/radiotherapy at the time were excluded), dementia or Alzheimer’s disease, acute or chronic liver disease, cerebrovascular disease, acute kidney injury, physical disability, coronary artery disease, heart failure, active infection, and those who were incompatible with ABPM measurement, aged <18 years and ≤80 years and active smokers were not included in the study.

### 2.3. Urinary Biomarkers and Proteinuria/Albuminuria

suPAR (Cat# E3759Hu) and N-terminal procollagen 3 propeptide (Cat# E1353Hu) concentrations were measured in 24 h urine samples using human-specific enzyme-linked immunosorbent assays (ELISA) (BT-laboratory, Shanghai, China) in accordance with the instructions of the manufacturer. suPAR assay sensitivity was 3.82 ng/L with inter-assay and intra-assay coefficients of variation of less than 10%. PIIINP assay sensitivity was 31.2 ng/L with inter-assay and intra-assay coefficients of variation of less than 10%. Measurements were performed on a Thermo Scientific Multiskan GO ELISA plate reader (Thermo Scientific, Waltham, MA, USA). The working principle of the ELISA kit is enzyme-linked immune sorbent analysis (ELISA) based on Biotin double-antibody sandwich technology. The sample was added to wells pre-coated with monoclonal antibody and then incubated. Biotin-labeled antibodies formed a complex with streptavidin-HRP. After incubation, the unbound conjugate was washed away. The color intensity formed after adding the substrate solution was measured at 450 nm and the concentrations were calculated using the calibration chart drawn in accordance with the standards. Protein and albumin concentrations in 24 h urine samples were determined using colorimetric methods on a COBAS 8000 (c702) biochemical analyzer (Roche Diagnostics GmbH; Mannheim, Germany).

### 2.4. Sociodemographic Data Survey

Demographic and epidemiological (gender, age and duration of disease) parameters, comorbid conditions (diabetes and hypertension), a history of nephrolithiasis, etiology in patients with Nx, and the number and group of antihypertensives were examined. Body mass index (BMI) was calculated as weight (kg)/height (m^2^).

### 2.5. Ambulatory Blood Pressure Monitoring and Carotis Intima Media Thickness (CIMT)

ABPM measurement was performed with a previously validated device [10,11] (Mobil-O Graph^®®^ (IEM, Stolberg, Germany)). Measurements were taken every 30 min during the day (06:00–21:59) and every 60 min at night (22:00–05:59). Appropriate-sized BP cuffs were used for ABPM. The daily routine activities of the patients were not restricted. Necessary information was given about sleep/wake schedules. BP-recorded readings exceeding 75% of the expected measurements were considered for analysis in accordance with the European Society of Hypertension guidelines [12]. SBP and DBP cut-off values were, respectively, an awake mean of 135≤ and/or 85≤, an asleep mean of 120≤ and/or 70≤, and a 24 h mean of 130≤ and/or 80≤. Classifications were made according to the degree of decrease in arterial blood pressure. The classifications were as follows: dipper: ≥10 to <20%, extreme dipper: ≥20%, nondipper: <10–0%, and riser: nocturnalBP > daytimeBP.

Carotid arteries were assessed with a 14 MHz linear probe with the patient in the supine position with a neck extension. B-mode, color and power Doppler ultrasound scans were performed. In addition, spectral flow volumes were evaluated. Bilaterally, CIMT values were evaluated in the common carotid artery from a 1 cm distance of bifurcation.

### 2.6. Statistical Analysis

For G Power analysis, the sample size was calculated using PASS software 11.0 (NCSS LLC, Kaysville, UT, USA). The minimum sample size required to test the correlation between 24 h arterial blood pressure measurement parameters and urinary type PIIINP and suPAR levels, with a significance level of 0.05 and 80% power under the one-way hypothesis test, 0.25 in the Ho hypothesis and 0.5 in the alternative H1 hypothesis, was calculated as 74. Considering the missing data, a total of 80 people was found to be sufficient.

The SPSS-22 program was used for statistical evaluation. Quantitative data were expressed as mean ± standard deviation or median (minimum–maximum), and qualitative data were shown as *n* (%). The compliance of the data with normal distribution was evaluated using the Kolmogorov–Smirnov test. Student’s *t* test and the Mann–Whitney U test were used for the comparison of the normally distributed and non-normally distributed data, respectively. The ANCOVA test was used for age adjustment in parametric data. The chi-square test was used to compare qualitative variables. Spearman’s correlation coefficient was used to evaluate the correlation analysis due to the concomitance of the non-normally distributed data. Backward multiple linear regression analysis was applied on correlated variables. A *p* value of <0.05 was considered significant.

### 2.7. Ethical Procedure

Permission was obtained from the local University Faculty of Medicine Health Sciences Ethics Committee, dated 1 June 2023 and allocated as no. 12/XIII. All procedures performed in human studies were carried out in accordance with the ethical standards of the institutional and/or national research committee, as well as with the Declaration of Helsinki (1964) and its later amendments or comparable ethical standards. Each patient was informed about inclusion in the study, and their consent was obtained.

## 3. Results

We evaluated 85 patients with SFK, aged between 19 and 80 years old, who were being monitored in the nephrology outpatient clinics at Muğla Sıtkı Koçman University Faculty of Medicine, Training and Research Hospital. We divided them into two groups: congenital and acquired. The basic demographic characteristics of the patients are shown in Table 1. Of the patients, 44 (51.8%) were female; 35 patients were Nx(+), and 50 patients were Nx(−). The most common etiological reason in Nx(+) patients was nephrectomy due to nephrolithiasis/pyelonephritis. The etiology of nephrectomy could not be clearly determined in 17%.

There was no difference between the two groups in terms of variables such as gender and BMI. Those with Nx(+) were significantly older. Of all the patients, 20 (23.5%) (*p* < 0.001) had a history of nephrolithiasis. There was no significant difference in terms of healthy kidney parenchymal thickness. The frequency of hypertension was similar in both groups.

Patients had a history of hypertension in 68.2% of cases and diabetes mellitus in 29.4%. When we examined antihypertensive treatments, 51.8% of the patients were using renin–angiotensin–aldosterone system (RAAS) inhibitors, 22.4% were using thiazides, 31.8% were using calcium channel blockers, 23.5% were using beta blockers and 2.4% were using alpha blockers.

Laboratory test results comparing the two groups are shown in Table 2.

The results were similar between groups. PIIINP and suPAR levels were higher in Nx(+) patients, and natriuria was higher in the Nx(−) group, although it was not statistically significant.

The correlation between age and GFR, some ABPM symptoms, BMI, CIMTs and the number of antihypertensives is shown in Table 3.

Newly diagnosed hypertension was detected in 4 of 27 patients (14.8%) who were not previously diagnosed with hypertension. High arterial blood pressure was detected in 34.5% of those under treatment with a diagnosis of hypertension. While arterial blood pressure was non-regulated in 25.7% of those with Nx(+), it was non-regulated in 30% of those with Nx(−).

The ABPM results and CIMT results of the patients according to the groups are shown in Table 4.

According to ambulatory systolic blood pressure measurements, among those with Nx(+), 28 patients (80%) were in the non-dipper (ND + RD) group and 7 patients (20%) were in the dipper group. Among those with Nx(Nx(−), 42 patients (84%) were in the non-dipper (ND + RD) group and 8 patients (16%) were in the dipper group. According to ambulatory diastolic blood pressure measurements, among those with Nx(+), 25 patients (71.4%) were in the non-dipper (ND + RD) group and 10 patients (28.6%) were in the dipper (dipper + ED) group. Among those with Nx(−), 33 patients (66%) were in the non-dipper (ND + RD) group and 17 patients (34%) were in the dipper (dipper + ED) group. There was no significant relationship between Nx(+) and Nx(−) patients in terms of ABPM and CIMT measurements, except for a higher mean SD in Nx(−) patients (Table 4).

There was a positive correlation between meanSyst and BMI and age, and a negative correlation between that and GFR decline (respectively, *p* = 0.004, r: 0.312; *p* = 0.012, r: 272; *p* = 0.008, r: −0.286). meanDiast was correlated with BMI and age (*p* = 0.025, r: 0.243; *p* = 0.038, r: 0.225, respectively). When we compared the two groups, in Nx(+) patients, there was a positive correlation between BMI and CRP and CRP/alb, and a negative correlation between that and alb/glob (*p* = 0.002, r: 0.507; *p* = 0.002, r: 0.511; *p* = 0.024, r: −0.380, respectively). In Nx(−) patients, there was a significant correlation between BMI and UA, CRP, homocysteine, proteinuria, albuminuria, meanSyst, 24 hMAP, pulse pressure, dSyst, dMAP, nSyst, nMAP and CRP/alb (*p* = 0.010, r: 0.361; *p* = 0.049, r: 0.280; *p* = 0.09, r: 0.365; *p* = 0.042, r: 0.289; *p* = 0.038, r: 0.295; *p* = 0.001, r: 0.444; *p* = 0.001, r: 0.480; *p* = 0.03, r: 0.380; *p* = 0.003, r: 0.409; *p* = 0.002, r: 0.439; *p* = 0.009, r: 0.368; *p* = 0.005, r: 0.401; *p* = 0.031, r: 0.306).

According to Nx status, PIIINP, suPAR and homocysteine correlations with other variables were observed and are shown in Table 5.

**Figure 1:**
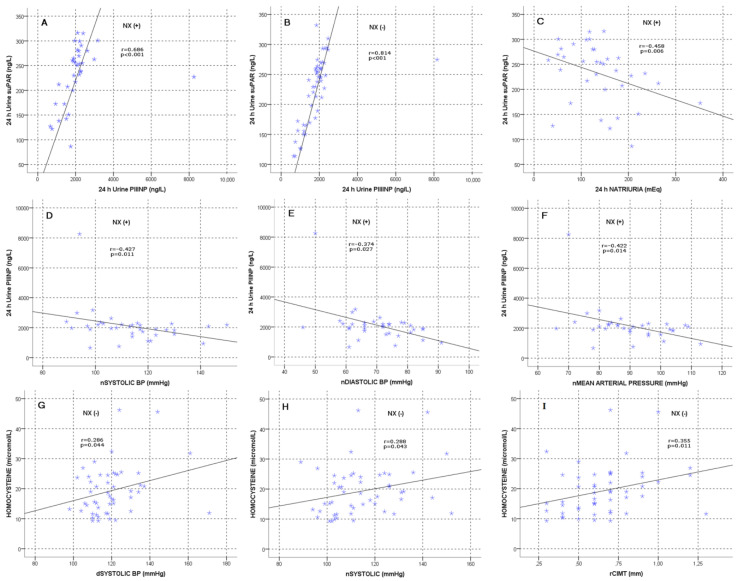
(**A**) Correlation between PIIINP and suPAR levels, Nx(+) group. (**B**) Correlation between PIIINP and suPAR levels, Nx(−) group. (**C**) Correlation between suPAR levels and natriuria, Nx(+) group. (**D**) Correlation between PIIINP and nSyst BP, Nx(+) group. (**E**) Correlation between PIIINP and nDiast BP, Nx(+) group. (**F**) Correlation between PIIINP and nMAP, Nx(+) group. (**G**) Relationship between homocysteine and dSyst BP, Nx(−) group. (**H**) Relationship between homocysteine and nSyst BP, Nx(−) group. (**I**) Relationship between homocysteine and rCIMT, Nx(−) group.

PIIINP levels and suPAR levels measured in 24 h urine showed a strong correlation in the same direction in both groups (*p* < 0.001). This was a finding we encountered for the first time in the literature. (Figure 1). While there was a significant negative correlation between PIIINP and meanSyst, nSyst, nDiast and nMAP in Nx(+) patients, this significance disappeared in Nx(−) patients. There was a negative correlation between suPAR and natriuria (*p* = 0.02 r: −0.251). While there was a significant negative correlation between suPAR and nDiast in those with Nx(+), the significance disappeared in those with Nx(−) (see Figure 1). When variables correlated with PIIINP were evaluated in the multiple linear regression analysis of Nx(+) patients, meanSyst (B = 57.28 [95% CI: 7.35–107.23], *p* = 0.026) and nORT (B = −107.70 [95% CI: −173, 51–41.90], *p* = 0.002) were found to be the main determinants. Additionally, when variables correlated with homocysteine were evaluated in multiple linear regression analysis in Nx(−) patients, eGFR (B = −0.130 [95% CI: −0.181–0.079], *p* < 0.001) and pulse pressure (B = 0.456 [95% CI: 0.252–0.660], *p* < 0.001) were found to be the main determinants.

In those with Nx(−), homocysteine had an inverse correlation with eGFR, albumin and Alb/Glob, and a significant correlation with BMI, UA, proteinuria, albuminuria, meanSyst, pulse pressure, dSyst, nSyst, CIMTr and the number of antihypertensives. In Nx(+) patients, there was only an inverse correlation with eGFR and Alb/Glob (Figure 1).

There was a significant negative correlation between Alb/glob and CIMTr and between neutrophil/platelet and CIMTr (*p* = 0.013 r: −0.269; *p* = 0.013 r: 0.270). There was a significant relationship between CRP/albumin and proteinuria, albuminuria, meanSyst, meanDiast, MAP, pulse, dSyst, dDiast, dMAP, nSyst, nDiast and nMAP (respectively, *p* = 0.0000, r: 0.425; *p* = 0.000, r: 0.407; *p* = 0.015, r: 0.263; *p* = 0.023, r: 0.247; *p* = 0.01, r: 0.285; *p* = 0.037, r: 0.226; *p* = 0.012, r: 0.272; *p* = 0.021, r: 0.251; *p* = 0.009, r: 0.290; *p* = 0.021, r: 0.250; *p* = 0.035, r: 0.229; *p* = 0.015, r: 0.271).

CRP showed a significant correlation with BMI (*p* = 0.001, r: 0.352), neutrophil count (*p* = 0.012, r: 0.272), proteinuria (*p* = 0.000 r: 0.395), albuminuria (*p* = 0.001, r: 0.366), natriuria (*p* = 0.041, r: 0.222), meanSyst (*p* = 0.022 r: 0.248), meanDiast (*p* = 0.34, r: 0.230), MAP (*p* = 0.019, r: 0.262) and pulse (*p* = 0.026 r: 0.241), and an inverse correlation with albumin (*p* = 0.014, r: −0.267).

There was a negative correlation between serum 25 OH Vitamin D and BMI, proteinuria, dMAP and natriuria/kaliuria (*p* = 0.042, r: −0.221; *p* = 0.023, r: −0.247; *p* = 0.046, r: −0.223; *p* = 0.014, r: −0.266). There was a negative correlation between 25 OH Vitamin D and natriuria (*p* = 0.033, r: −0.361) and natriuria/kaliuria (*p* = 0.035, r: −0.357) in those with Nx(+), whereas there was a negative correlation between 25 OH Vitamin D and dMAP (*p* = 0.021 r: −0.336) in those with Nx(−).

There was a significant correlation between natriuria and proteinuria (*p* = 0.000, r: 0.407), albuminuria (*p* = 0.014, r: 0.265) and diastolic SD (*p* = 0.03, r: 0.236).

Serum uric acid (UA) levels had a significant correlation with homocysteine (*p* = 0.000, r: 0.420), proteinuria (*p* = 0.016, r: 0.260), albuminuria (*p* = 0.001, r: 0.364) and CIMTr (*p* = 0.017, r: 0.257), and an inverse correlation with GFR (*p* = 0.001, r: −0.352). In Nx(+) patients, UA was inversely correlated only with alb/glob (*p* = 0.021, r: −0.388). In those with Nx(−), UA was significantly correlated with BMI (*p* = 0.01, r: 0.361), homocysteine (*p* = 0.001, r: 0.469), proteinuria (*p* = 0.024, r: 0.318), albuminuria (*p* = 0.001, r: 0.438), CIMTr (*p* = 0.043, r: 0.287) and CIMTl (*p* = 0.037, r: 0.295), and inversely correlated with GFR (*p* = 0.000, r: −0.538).

The BMI mean value was 28.3197 ± 4.69408 kg/m^2^. When we look at its relationship with other parameters, there was a significant correlation with age (*p* = 0.006, r: 0.296); CRP (*p* = 0.001, r: 0.352); proteinuria (*p* = 0.007, r: 0.292); meanSyst (*p* = 0.004, r: 0.312); meanDiast (*p* = 0.025, r: 0.243); MAP (*p* = 0.001, r: 0.367); dSyst (*p* = 0.008, r: 0.285); CIMTl (*p* = 0.026, r: 0.242); and the number of antihypertensives (*p* = 0.016, r: 0.261). It was inversely correlated with GFR (*p* = 0.011, r: −0.274) and 25 OH Vitamin D (*p* = 0.042, r: −0.221).

## 4. Discussion

### 4.1. General Perspective

During the nephrogenesis process, renal anomalies may occur due to genetic changes, environmental factors and nutritional effects. The frequency of urogenital anomalies is approximately 1–3/500 live births. This accounts for 40–50% of pediatric CKD cases [13]. In unilateral renal parenchymal anomalies under the heading of CAKUT, there is a decrease in kidney mass and nephron number. This group consists of unilateral renal hypoplasia, dysplasia, hypodysplasia, multicystic dysplastic kidney and renal agenesis. URA and multicystic dysplastic kidney are the two most common parameters in the etiologies of SFK [14]. In adults, CAKUT is responsible for approximately 7% of end-stage renal disease (ESRD) globally [15].

As the number of functioning nephrons decreases in patients with SFK due to both acquired and congenital causes, physiological adaptive changes secondary to renal compensation—such as an increase in single-nephron GFR, an increase in filtration fraction, an increase in intra-glomerular pressure and an increase in renal blood flow—can occur. These changes are referred to as the hyperfiltration response. The aim is to maintain a stable GFR for a while, but in the long run, as a result of these hemodynamic changes, varying degrees of glomerulosclerosis, proteinuria, hypertension and chronic kidney damage develop in the kidneys. In this context, Srivasta et al. demonstrated in rats that glomerular hyperfiltration leads to an increase in fluid flow shear stress (FFSS) on podocytes in conditions characterized by a decrease in the number of nephrons. They emphasized that FFSS also potentiates podocyte damage and CKD progression [16].

### 4.2. SFK and Renal Dysfunction Findings

CKD is a progressive, irreversible clinical condition that can lead to many serious complications throughout life, especially cardiovascular abnormalities. According to 2019 reports, the number of deaths caused by CKD was 1.4 million (11th) and the percentage of deaths due to CKD among all the causes of death was 2.5%. Patients with SFK are considered to be in the risk group for CKD due to the frequent occurrence of hypertension, especially in the long term, and due to the pathophysiological conditions that may occur in a solitary kidney. Sanna-Cherchi et al.’s clinical follow-up study on this subject is very important in terms of taking this risk into consideration. The study showed that 20–50% of 312 children who had SFK (only patients with CAKUT) in childhood developed ESRD in their third decade [17]. In our patients, the rate of patients with GFR < 60 mL/minute/m^2^ was 37%. In total, 72% of the patients had < 150 mg/day of proteinuria; 30–300 mg/day of albuminuria in 24 h urine was detected in 23 patients; and 300 mg/day < of albuminuria was detected in 23 patients. In total, 68.2% patients had hypertension. Compared to other series, the frequency of hypertension and proteinuria/albuminuria was higher in our study population. Oldrizzi et al. found that the prevalence of hypertension was 40%, the prevalence of proteinuria was 46% and the prevalence of renal dysfunction was 15% in 39 patients with URA, with a mean age of 33 years [18]. In another study, hypertension was detected in 36.9%, proteinuria was detected in 35.4%, and both hypertension and proteinuria were detected in 20% of 65 congenital solitary kidney patients with an average age of 37 years [19]. Argueso et al. showed that out of 37 patients with URA at an average age of 37 years, proteinuria developed in 19%, 47% of 47 patients had hypertension and 13% of 32 patients had renal dysfunction [20]. We can attribute this high prevalence in our study to the limited use of RAAS inhibitors (only around 50% of patients were using them). It has been shown that in patients with solitary kidneys, RAAS blockade can reduce the progression of renal lesions in the intact kidney. The inhibition of macrophage and myofibroblast proliferation and improvements in glomerular and tubular lesions have been demonstrated in rats with the use of high doses of ACE-inhibitors [21]. However, it is known that there is resistance/reluctance in real life to use RAAS inhibitors in patients with SFK, especially in the non-nephrological approach. Moreover, although we cannot document it, according to our observations, we can interpret this as the fact that patients, especially those diagnosed incidentally with SFK, attend check-ups more reluctantly and irregularly after overcoming the ‘shock of the first diagnosis’. Similar progressive processes can be mentioned for the acquired group. In a meta-analysis of seven general population-based cohorts, Grams et al. found a 3.5- to 5.3-fold higher risk of developing ESRD among living kidney donors compared with that of age-matched controls, although the absolute risk after unilateral Nx was low [22]. A significant fraction of the acquired SFK group consists of living kidney donors. Although their selection from ‘healthy’ volunteers seems to be beneficial in terms of long-term chronic diseases, there is a risk of long-term renal damage due to the SFK-related renal risks mentioned and discussed above. A recently published meta-analysis revealed a current risk of developing ESRD (HR of 5.57 (95% CI: 2.03–15.30)) in living kidney donors compared to the control group (general group and healthy group) [23]. In another study in which 338 living renal donors were evaluated retrospectively, new hypertension was detected in 16.8% of patients and stage 3 CKD development was detected in 14.7% of patients during follow-up. Being older at the time of transplantation and having low eGFR risk factors were found to be significant in the development of CKD [24]. In a large cohort study examining more than 1000 renal donors over a period of approximately 8 years, comparing it with that of the healthy blood donors, an increased risk of hypertension was observed (SIR, 1.40;95% confidence interval (1.17–1.66)) [25].

In the current approach, in SFK patients, in addition to traditional renoprotective approaches, there is a need for markers based on biochemical or technical measurement methods that have predictive potency and can detect possible differences according to SFK etiologies.

Hyperfiltration is the main player at the beginning of the scenario that includes all abnormal glomerular changes in patients with SFK. According to the study of Cachat et al., urinary albumin showed a weak relationship with hyperfiltration markers in patients with SFK (19% was Nx(+)), and it was suggested that factors other than glomerular hyperfiltration may contribute to the development of CKD [26]. In Srivastava et al.’s study including cSFK patients, urinary Prostaglandin E2 (PGE2) was evaluated as a biomarker of the early effects of damage caused by hyperfiltration before the development of microalbuminuria in individuals with SFK. They showed that an increase in urinary PGE2 precedes the development of severe albuminuria [27]. In another study, Naik et al. focused on the role of IGF-1 in the compensatory growth of the remaining kidney after unilateral Nx. They stated that hyperfiltration after unilateral Nx is associated with an increased filtration of IGF-1 (exposure to growth factors), which triggers glomerular expansion and leads to critical podocyte depletion, followed by proteinuria and glomerulosclerosis [28].

### 4.3. Congenital and Acquired SFK, Not the “Same Script, Different Cast”?

Acute reduction—such as nephrectomy—in kidney mass has a variable effect on renal function compared with that under congenital etiologies. The most important point here is the timing of the decrease in renal mass. There may be significant differences in the expression of renal pathologies between those with congenital and acquired SFK; while cSFK still has the potency of ongoing new nephron formation, nephrogenesis is stopped during Nx in aSFK. This finding may imply a higher sensitivity to significant glomerular hyperfiltration in those with aSFK [29]. Sudden massive nephron loss compensated for by glomerular hypertrophy is a precursor to progression to glomerulosclerosis and progressive renal injury. A congenital single functioning kidney may be less hypertrophic compared to an acquired single kidney. An adaptive response to congenital nephron deficiency is possible at an early fetal age, when the number of glomeruli may still increase due to active mitosis. More nephrons means less glomerular hyperfiltration, and thus a lower incidence of glomerulosclerosis [30].

Spira et al. argued that one of the main differences between congenital/acquired SFKs that aSFKs is that the latter have a lower number of nephrons to compensate for hyperfiltration and hypertrophy than in congenital conditions [31]. Surgical removal of kidney tissue prompts the remaining nephrons to immediately undergo compensatory changes to quickly overcome the significant loss in filtration surface area. Given that mature glomeruli have low mitotic activity, the remaining nephrons will adapt by increasing only their size and not their number, ultimately reaching a maximum level of hypertrophy beyond which a compensatory reaction can no longer be achieved. Studies on renal functional reserve in patients with aSFK have frequently shown a partial or complete blunting of the response several years after nephrectomy. Therefore, aSFK may possibly be more vulnerable to additional stress than is cSFK.

Lenihan et al. showed that glomerular hyperfiltration was not accompanied by a significant increase in glomerular pressure in adult kidney donors. Similarly, it was shown that glomerular capillary pressure increased minimally in adult rats. In contrast, nephrectomy in newborn guinea pigs, where nephrogenesis ceases before birth, resulted in a 30% increase in glomerular filtration pressure [32,33]. These differences in results suggest that a loss of kidney mass early in life leads to a greater risk of kidney and cardiovascular disease than that with the loss of kidney mass later in life.

In various animal models where kidney mass decreases during nephrogenesis, the number of nephrons in the SFK increased by 4% to 50%. Larsson et al. showed that when renal mass decreases during active nephrogenesis in rats, the level of glomerular hyperfiltration doubles compared to that later in life [34]. Although it is not currently possible to determine the number of nephrons in living individuals, Maluf reported that both kidney weight and nephron numbers were increased in a single case [35].

In a large cohort study of over 270,000 young to middle-aged individuals without CKD at the baseline and with an average follow-up of nearly 5.5 years, SFK was independently and modestly associated with an increased risk of CKD, and CKD risk was higher in those with aSFK than in those with cSFK [6]. Similarly, Jaoude et al. reported that there was an inverse relationship between GFR and follow-up time in patients with aSFK, but there was no such relationship in patients with cSFK. It has been suggested that the adaptive response following the decrease in renal mass may begin much earlier (near the 22nd week) in cSFK patients than in aSFK patients, and that aSFK patients may have worse functional adaptation since mature glomeruli have low mitotic activity [36]. These different findings show us that different pathogenetic mechanisms may play a role in congenital and acquired SFK.

The KIMONO study also found that SFK due to acquired kidney loss in childhood was associated with a higher incidence of kidney damage compared with that under congenital loss [29]. In Yazıcı et al.’s study, eGFR values were lower in patients with reduced renal mass due to nephrectomy than those in patients with congenital solitary kidneys and unilateral hypoplastic nonfunctioning kidneys [37]. The reason for this difference in eGFR values may be that patients with congenital renal mass reduction have a single kidney for a much longer time than do patients with nephrectomy, and GFR values are probably preserved due to long-term adaptation mechanisms. In their study, Kasap-Demir et al. showed that patients with cSFK maintained better GFR levels by developing better adaptation mechanisms [38].

Gadalean et al. showed that in patients with congenital renal mass reduction, adaptive tubular changes occur earlier than they do in those with acquired ones, thus leading to better renal outcomes [39]. Bohle et al. noted that patients with acquired single kidneys showed higher levels of urinary tubular biomarkers compared to those of patients with congenital single kidneys, which is indicative of tubular damage [40].

Homocysteine may not be the same potential risk factor between aSFK and cSFK patients. Homocysteine is a non-proteinogenic, sulfhydryl-group-containing, non-essential intermediate amino acid formed during the conversion of methionine, a sulfur-containing, essential amino acid, into cysteine. As an established risk factor for hypertension, hyperhomocysteinemia is positively associated with blood pressure. Fu et al. found that the average homocysteine concentration was higher in hypertensive patients than that in non-hypertensive patients, and revealed a reasonable relationship between high homocysteine levels and an increased risk of hypertension [41]. It has been suggested that a 5 µmol/L increase in circulating homocysteine concentration would increase systolic and diastolic blood pressure by 0.5–0.7 mmHg and 0.7–1.2 mmHg, respectively [42]. It is thought to cause abnormalities such as endothelial damage, the induction of inflammation and cell death, a decrease in nitric oxide levels, increased concentrations of reactive oxygen species and oxidative stress, cellular methylation abnormalities and abnormal lipid metabolism [9,43]. It has been reported that hyperhomocysteinemia potentiates cardiac inflammation and fibrosis by increasing both itself and AngII-induced NF-κB p65 and TGF-β activation [44]. Endothelial homocysteine has been shown to significantly impair shear-stress-induced arterial dilatation and nitric oxide release in Wistar rats, which triggers vascular superoxide-mediated angiotensin II signaling through Ang II receptor 1 [45,46].

Homocysteinemia can occur in the early stages of CKD and increases with the progression of kidney damage. The positive correlation of the plasma homocysteine level with the creatinine level has been shown in one study [47]. In our study, homocysteine was compatible with negative renal and cardiovascular risk factors in Nx(−) patients. Although there was no difference in GFR between the Nx(+) and Nx(−) groups and although age was statistically lower in the Nx(−) group, homocysteine was correlated with BMI and UA as a marker of inflammation in the Nx(−) group. Additionally, this correlation continued with the proteinuria/albuminuria level and CIMT. It was interesting that this significant correlation, which appeared in Nx(−) patients, disappeared in Nx(+) patients. We think that this may have been caused by a longer chronic inflammatory process in Nx(−) patients than that in acquired patients.

In one study, homocysteine was associated with a non-dipping pattern, a strong predictor of cardiovascular mortality and morbidity in hypertensive individuals [48]. Although there was no significant correlation with dipping status in our study, its correlation with nSyst supported this. Kasiske et al. compared renal donors with healthy controls in a prospective, controlled study. Although the decrease in GFR and the increase in homocysteine and UA levels were found to be significantly higher in renal donors at the 36-month follow-up, no difference was detected between both groups in ABPM measurement follow-ups [49]. In the study of Carnagarin et al., homocysteine was found to be compatible with pulse wave velocity, which is an important cardiovascular risk factor [48]. With all these findings, the question arises of whether or not the endothelial damaging effect of homocysteine may be more potent in Nx(−) patients with SFK than it is in Nx(+) patients.

### 4.4. Our Findings about ABPM

ABPM is a simple, non-invasive examination that can be used to monitor a patient’s blood pressure at different time points over a 24 h period, as well as to detect the circadian rhythm of blood pressure, blood pressure variability and morning peak blood pressure. Many studies have shown that a higher BP in ABPM is a stronger predictor than is office BP in predicting target organ damage and CVD events [50]. In the 2021 United States Preventive Services Task Force reports on hypertension screening, ABPM was recommended as the reference standard for out-of-office BP monitoring and for confirming the initial diagnosis of hypertension [51].

In healthy conditions, arterial blood pressure exhibits a circadian rhythm, with blood pressure falling by 10% to 20% during nocturnal sleep due to a decrease in sympathetic tone and a parallel increase in vagal activity during the sleep period. This nighttime decrease is called normal dipping (Dipper pattern). When the nocturnal drop in blood pressure during sleep becomes blunted or disappears (<10% of the daytime blood pressure level), this is called non-dipping (NonDipper pattern). When nocturnal blood pressure during sleep rises above awake levels, this is called reverse dipping (Reverse Dipper/Risers). When the nocturnal drop in blood pressure is severe (>20% of the daytime blood pressure level), this is called extreme dipping (ED pattern). In the ESH/ESC Hypertension 2023 guideline, ABPM/HBPM is recommended with strong evidence for the evaluation of nocturnal hypertension (especially in risk groups such as CKD) and monitoring when evaluating treatment strategies in CKD patients, since the ND pattern or increased nocturnal BP is common [4].

Night values of SBP ≥ 120 mmHg or DBP ≥ 70 mmHg were considered nocturnal hypertension in most studies. Isolated nocturnal hypertension (with normal daytime pressure) has been shown to have a significantly higher risk of cardiovascular events compared to that under normotension, while another study has also shown it to be associated with hypertension-induced target organ damage [52,53]. Vinyoles et al. demonstrated the independent prognostic importance of nighttime systolic blood pressure and 24 h BP determined via ABPM at the end of a 6.6-year follow-up in patients without previous cardiovascular disease [54]. A study of 8711 participants found that individuals with untreated isolated nocturnal hypertension had a significantly higher risk of total death and cardiovascular events compared to normotensives [55]. In one study (mean age 47.0 ± 11.7 years), it was stated that nighttime systolic BP was a more specific determinant of arterial stiffness in the evaluation performed with PWV in young and middle-aged adults with non-dipper hypertension [56]. When we evaluated night blood pressure parameters in our study, there was a reverse correlation between PIIINP and nSys, nDia and nMAP in Nx(+) patients. This significance was lost in those with Nx(−). PIIINP levels may be a biomarker that can be used to predict night blood pressure in this patient group. In the Nx(+) group, the suPAR level also had an inverse correlation with nDiast. There was also a positive correlation between nSyst and homocysteine in the Nx(−) group. The relationship with these biomarkers can be taken into consideration when evaluating nocturnal blood pressure parameters, which are strong indicators of cardiovascular and renal endpoints.

In our study, the serum homocysteine level did not generally correlate with ABPM measurements. In addition, although there was no significant difference in concentration between the Nx(+) and Nx(−) groups, there was a significant positive correlation with mSys, PP, dSys and nSYS in Nx(−) groups. This correlation was not present in those with Nx(+). In addition to this significant relationship in hypertension parameters, pulse pressure was also significantly higher in these patients. High ambulatory pulse pressures have also been shown to be associated with cardiovascular morbidity and mortality [57]. Current evidence suggests that hypertension is accompanied by a chronic pro-inflammatory state and that inflammation may play a role in both the development of hypertension and end organ damage. Ciobanu et al. reported that a high serum E-selectin level, as an inflammatory marker, was associated with daytime and 24 h diastolic BP variability in patients with type 2 diabetes [58]. In Kayıkçıoğlu et al.’s study, the CRP/albumin ratio was found to be significant in predicting non-dipper patients even in normotensive individuals, and inflammation was emphasized [59]. In our study, CRP/albumin, as an inflammation indicator, was unrelated to dipper status, but had a significant correlation with both proteinuria/albuminuria and other ambulatory results (MAP, pulse, nMAP, etc.).

uPAR is a membrane receptor protein produced in the podocytes, endothelium and immature myeloid cells. Activated uPAR can be degraded and detected in systemic circulation in its soluble form, suPAR (glycosyl-phosphatidyl inositol anchored 3-domain membrane protein). uPAR/suPAR can cause glomerular damage in podocytes via the αvβ3 integrin pathway [60]. Concentrations of suPAR in serum and urine are considered to reflect endothelial dysfunction and immune and inflammatory activation.

Systemic chronic inflammation is a central problem for many diseases and clinical conditions. suPAR is a potential biomarker that reflects the state of chronic inflammation (especially vascular) and is relatively stable (less inducible than CRP, without major changes in blood levels) [61]. It is possible that there may be some clinical consequences caused by the inflammatory process in patients with SFK. We think that there may be differences in subclinical inflammation between patients with c/aSFK. In our study, we showed the relationship between homocysteine and inflammatory markers such as UA, CIMT, UA and alb/glob in Nx(−) patients. Although they did not reach statistical significance, CRP, UA and CRP/Alb ratios were higher and 25 OH Vitamin D was lower in Nx(−) patients. These findings were not accompanied by changes in 24 h urinary suPAR levels. The fact that the plasma serum suPAR level was not measured in our study may be a reason for this. In the literature, plasma/serum suPAR levels have been measured much more frequently than urinary suPAR has been (usually suPAR/creatinine in morning spot urine). In our study, we investigated the level of suPAR in 24 h urine from a different perspective. As far as we know, there is no study in the literature examining suPAR levels in 24 h urine.

There are conflicting publications regarding the predictive potency of suPAR in CKD progression [62,63]. In a large cohort study including 3683 patients, higher suPAR levels were associated with CKD progression [64]. Rowaiye et al. reported that plasma suPAR was inversely correlated with eGFR, but no correlations were present between urine suPAR/creatinine ratio and eGFR [65]. Fujimoto et al. found no significant differences among patients with FSGS, MCNS and MN in urinary suPAR levels in the pretreatment period. Therefore, they postulated that the difference between these entities may be due to molecular size or the glycosylation of suPAR, possibly causing differences in the renal histological phenotype [66].

Functional adaptations secondary to a decrease in renal mass include intrarenal vasodilation, leading to increased glomerular capillary pressure and hyperperfusion injury to the glomeruli. This process leads to glomerular cell proliferation, the infiltration of macrophages, and the progressive accumulation of components such as fibronectin, collagen type I and type III, and decorin. This non-immunological process can lead to damage being sustained to podocytes and the glomerular microvasculature, leading to proteinuria, widespread fibrosis and a progressive deterioration in renal function. In a normal kidney, interstitial type III collagen expression is small in proportion; however, in scarred kidneys, the expression of collagen type III increases in the interstitium during the earliest stages of fibrosis, and PIIINP accumulates in sclerotic glomeruli. During the synthesis and deposition of type III collagen, an amino terminal pro-peptide of type III collagen, called the N-terminal pro-peptide of type III collagen, is produced. During the processing of pro-collagen prior to its deposition in the ECM, a portion of PIIINPs are cleaved and released into the ECM and fluids, including blood and urine.

El Ghoul et al., in their prospective, non-SFK study of 199 individuals, showed an inverse correlation between UPIIINP/Cr and eGFR and a significant correlation with renal fibrosis [67]. In a study evaluating early Fabry fibrosis markers, it was shown that the level of PIIINP (along with other urinary peptides) was increased in the early stage of the disease (especially in cardiomyopathy) [68].

In our study, unlike in the literature, we examined PIIINP levels in 24 h urine, not in spot urine samples. We could not show a relationship with GFR decline, but a significant inverse correlation with nighttime ABPM measurements and meanSyst in the Nx(+) group was a finding encountered for the first time in the literature. In the study by Söylemezoğlu et al., although urinary and serum PIIINP levels showed a strong histopathological correlation with the degree of renal fibrosis, no correlation was found between them and creatinine values [69]. Although there are many studies in the literature that emphasize the relationship between hypertension and PIIINP, there are also studies on the contrary that do not detect any significance. In their study on patients without a previous cardiac pathology, Dhingra et al. did not find any relationship between plasma PIIINP and incidental hypertension or progression in the blood pressure category [70]. Timms et al. reported that there was no difference in plasma PIIINP levels between hypertensive and normotensive patients [71]. More studies are needed to examine in detail the relationship between PIIINP and hypertension in patients with SFK.

Increased CIMT is associated with higher blood pressure and an increased left ventricular mass index. Its predictive significance for the risk of developing atherosclerosis and cardiovascular disease is well established. In our study, CIMT showed a relationship with variables such as age, BMI and UA, as expected. In group analyses, there was no significant difference between Nx(+)/Nx(−). However, in Nx(−) patients, homocysteine was correlated with CIMT. This significance in terms of atherosclerotic clinical outcomes in Nx(−) patients may be a guide for future studies.

As shown in a retrospective study, BMI was likely a factor associated with the progression of renal function loss in patients with a single kidney [72]. In a population-based, comprehensive study by Alfandary et al., BMI (OR 1.093, 95% CI: 1.092–1.095) was associated with increased kidney damage in adolescents with cSFK, and the higher the BMI category, the higher the renal damage [73]. In our study, BMI was significantly associated with possible renal and cardiovascular adverse effects such as UA, proteinuria/albuminuria, homocysteine and ambulatory hypertension findings (24 hMAP, pulse pressure, nMAP, etc.), especially in Nx(−) patients, unlike the case for Nx(+) patients. This significance was not detected in Nx(+) patients. The fact that the negative metabolic effects of BMI are more pronounced in those with Nx(−) may necessitate more awareness about targeting the ideal weight in this patient group. Similarly, as an inflammation indicator, UA in those with Nx(−) had a significant inverse correlation with BMI, homocysteine, proteinuria, albuminuria, CIMT and GFR, unlike those with Nx(+). These similar findings again suggested the inflammatory potency between variables, especially in Nx(−) patients.

As a result, with the improvement in medical conditions, more and more children with congenital renal disease are surviving and reaching adulthood. However, the number of renal donors is also increasing. This shows us that we will encounter possible nephrological complications in patients with SFK more frequently. Hypertension target values should be determined especially in this patient group and should be included in hypertension guidelines. In line with the current literature, it seems rational for ABPM to become a priority in hypertension screening and monitoring treatment response in patients with SFK. In our study, the high rate of patients who were aware of their hypertension or whose course was unregulated even while under antihypertensive treatment supports this. Our study also showed that although there is no difference in ABPM findings between patients with Nx(+) and Nx(−), there may be various differences in markers that correlate with ABPM findings.

Our study has some limitations. In terms of patient selection, our hospital is the main tertiary referral hospital in our region. Almost all SFK patients (with or without nephrectomy) are referred to our nephrology out-patient clinic (from other clinics/hospitals; primary care centers). We have many SFK patients in our routine follow-up list. Hence, in accordance with the inclusion/exclusion criteria, we selected these patients. Furthermore, regression analysis results may be of limited value. The number of patients in our study was small. We think this is due to the broad scope of inclusion and exclusion criteria. Additionally, it was a disadvantage that it was a single-center study. We could not provide an etiological explanation for six patients with nephrectomy due to lack of documentation. Another limitation was that there was also no widely accepted consensus on GFR measurement in patients with SFK [74,75]. In our study, suPAR and PIIINP levels were measured in 24 h urine. We could not find any study in the literature that included suPAR levels measured from a 24 h sample. For PIIINP, we saw that only one publication used a 24 h sample. In this publication, the urinary excretion of PIIINP correlates with the degree of interstitial fibrosis, but not with albuminuria or reduced GFR and serum concentration of PIIINP [7]. Due to this situation in the literature, we had difficulty making comparisons. Another point that limited the study was that plasma/serum suPAR and PIIINP were not measured simultaneously, and urine PIIINP/creatinine was not measured simultaneously. Moreover, using the nephrectomy group as the comparator group for the solitary kidney group is another limitation; however, the renal outcomes of patients with SFK are still debated among nephrologists. Patients who have undergone nephrectomy may have a different risk for kidney injury than do patients with a solitary functioning kidney. Our main purpose in this study was to discuss and speculate about the possible different effects of nephrectomy in patients with SFK. Therefore, we analyzed the renal outcomes in patients with SFK and compared factors associated with fibrosis, inflammation and blood pressure results in the cSFK and aSFK groups. Due to etiological diversity, it was not possible to completely avoid heterogeneity, even partially, in creating and comparing subgroups. It is still debated whether being born with a solitary kidney or losing a kidney after birth would have a differential effect on final renal outcomes, and large comparative studies are needed on this subject.

### 4.5. Directions for Future Research

It would be informative if future studies on the population of SFK patients are able to predict inflammatory, fibrotic and cardiovascular risk markers among different etiologies of this group. Studies to develop preventive strategies for long-term complications in this patient group are important. Developing similar guidelines for adults as well as pediatric patients may be instructive [76]. The phosphatase and tensin homolog (PTEN) is a negative regulator of protein kinase B and the mammalian target of the rapamycin pathway. In addition to being a tumor suppressor gene, it is also associated with other molecular and clinical conditions such as cellular migration, association with peripheral insulin resistance, and diabetic nephropathy [77]. In a study conducted by An et al. on rats, the possible role of PTEN deficiency in the pathophysiology of hypertension-associated renal fibrosis was emphasized. It was shown that Angiotensin II-related renal damage (proteinuria, renal fibrosis) is significantly increased in PTEN knockout mice, independent of the level of hypertension [78]. Simeoni and colleagues emphasized the role of RAAS activation in the pathogenesis of the solitary kidney [30]. In this context, we think that PTEN can be used as a biomarker of fibrosis and renal damage in future studies in patients with SFK. With the increasing number of RCC patients (due to the possible need for Nx) and the number of renal donors, we will encounter more patients with solitary kidneys in the future; therefore, identifying patients at increased risk of kidney damage and GFR reduction should be the most important goal in this patient group. In addition to traditional risk factors, the predictive values of new biomarkers regarding prognosis will become increasingly important. There is a need for more comprehensive studies that homogeneously compare patients with SFK by grouping them etiologically.

## 5. Conclusions

Nx(+) or Nx(−) status may change the clinical approach when evaluating patients with SFK. To the best of the authors’ knowledge, although our study population is small, it is the first study to evaluate the correlations of ABPM measurements with biomarkers such as 24 h urinary PIIINP, serum homocysteine and 24 h urinary suPAR by grouping patients with SFK as Nx(+)/Nx(−). Serum homocysteine levels may play a key role in the renal and cardiovascular outcomes of patients with cSFK and aSFK.

## Figures and Tables

**Table 1:** Demographic and basic findings.

	Nx(+) (*n* = 35)	Nx(−) (*n* = 50)	*p*
Age	56.88 ± 13.91	45.86 ± 13.47	<0.001
f/m (*n*, %)	19 (54.3%)/16 (45.7%)	25 (50.0%)/25 (50.0%)	0.697
BMI (kg/m^2^)	29.42 ± 4.63	27.54 ± 4.62	0.347
Parenchyma thickness, SFK (mm)	14.48 ± 2.88	15.41 ± 3.27	0.895
SFK, (right%)	24 (68.6%)	25 (50.0%)	0.088
Presence/history of nephrolithiasis (*n*, %)	11 (31.4%)	9 (18.0%)	0.151
Hypertension (*n*, %)	24 (68.6%)	34 (68.0%)	0.956
Diabetes Mellitus (*n*, %)	13 (37.1%)	12 (24.0%)	0.191
Smoking (*n*, %)	11 (31.4%)	20 (40.0%)	0.419
LDL < 130 mg/dL (*n*, %)	28 (80.0%)	35 (70.0%)	0.300
Mean antihypertensives, (*n*)	1 (0–4)	1 (0–4)	0.978
Antihypertensive treatments			
RAAS inhibitors (*n*, %)	16 (45.7%)	28 (56.0%)	0.350
Thiazides (*n*, %)	7 (20.0%)	12 (24.0%)	0.663
Calcium channel blockers (*n*, %)	11 (31.4%)	16 (32.0%)	0.956
Beta blockers (*n*, %)	10 (28.6%)	10 (20.0%)	0.359
Alpha-blockers (*n*, %)	2 (5.7%)	0 (0.0%)	0.167
Nx etiology			
Renal cancer	9 (25.7%)		
Nephrol./pyelonephr.	10 (26.6%)		
Unknown	6 (17.1%)		
Donor	7 (20.0%)		
Atrophic/dysfunction	2 (5.7%)		
Hydronephrosis	1 (2.9%)		

Results are presented as *n* (%), mean ± standard deviation or median (minimum-maximum). Age adjustment was performed for parametric data.

**Table 2:** Laboratory results.

	Nx(+)	Nx(−)	*p*
Creatinine (mg/dL)	1.15 (0.48–4.00)	1.08 (0.54–4.23)	0.780
GFR (mL/min/1.73 m^2^)	64.64 ± 26.41	72.28 ± 31.91	0.481
UA (mg/dL)	5.91 ± 1.08	6.16 ± 1.76	0.358
CRP (mg/dL)	1.63 (0.60–23.15)	2.92 (0.60–63.30)	0.188
Albumin (g/dL)	4.53 ± 0.37	4.56 ± 0.31	0.906
25OH Vitamin D (ng/mL)	21.12 ± 11.21	18.45 ± 9.56	0.736
Hemoglobin (g/L)	13.38 ± 2.11	13.60 ± 1.80	0.607
Homocysteine(µmol/L)	16.60 (7.90–41.20)	18.05 (9.30–46.20)	0.151
Proteinuria (24 h, mg/d)	210 (44–4808)	250 (38–6829)	0.448
Albuminuria(24 h, mg/d)	23 (2–2649)	48.5 (4–5615)	0.262
Natriuria (24 h, mmol/d)	138.11 ± 68.74	173.68 ± 87.68	0.156
Kaliuria (24 h, mmol/d)	45.04 ± 20.58	51.19 ± 21.74	0.460
suPAR (24 h urine, ng/L)	250.70 (86.40–316.30)	239.85 (113.70–331.90)	0.422
PIIINP (24 h urine, ng/L)	1988.00 (661.30–8254.00)	1869.50 (652.10–8165.00)	0.80
Natriuria/kaliuria	3.13 (0.95–7.70)	3.23 (1.23–7.27)	0.526
CRP/albumin	0.36 (0.12–5.35)	0.66 (0.12–13.33)	0.203

Results are presented as mean ± standard deviation or median (minimum-maxiumum). Age adjustment was performed for parametric data.

**Table 3:** Significant correlations with age parameter.

Parameter	r	*p*
Age (years) with:		
BMI (kg/m^2^)	0.296	0.006
eGFR (mL/min/1.73 m^2^)	−0.507	<0.001
meanSyst	0.272	0.012
24 hMAP	0.280	0.012
dSyst	0.238	0.028
dMean	0.229	0.041
nSyst	0.294	0.006
nMean	0.280	0.012
CIMTr (mm)	0.501	<0.001
CIMTl (mm)	0.602	<0.001
Antihypertensives	0.354	0.001

**Table 4:** Ambulatory blood pressure results, CIMTs.

	Nx(+)	Nx(−)	*p*
24 h-Mean Syst.	118.22 ± 15.20	118.24 ± 12.96	0.379
24 h-Mean Diast.	74.77 ± 9.53	75.94 ± 9.25	0.262
24 h-MAP	94.72 ± 11.74	75.94 ± 9.25	0.254
24 h-Pulse	72.97 ± 9.05	76.92 ± 9.69	0.318
24 h-Pulse Pressure	43.59 ± 10.00	42.30 ± 8.00	0.922
Mean SD	9.22 ± 2.51	10.15 ± 3.24	0.037
DaytimeSyst.	119.91 ± 15.79	119.62 ± 13.57	0.496
DaytimeDiast.	76.17 ± 9.78	77.40 ± 9.89	0.306
DaytimeMAP	96.24 ± 12.22	96.74 ± 11.15	0.359
NighttimeSyst.	113.25 ± 15.04	114.36 ± 14.72	0.175
NighttimeDiast.	70.34 ± 10.23	72.10 ± 10.76	0.186
NighttimeMAP	89.90 ± 11.84	91.72 ± 12.03	0.110
CIMTr (mm)	0.73 ± 0.29	0.65 ± 0.23	0.852
CIMTl (mm)	0.76 ± 0.27	0.66 ± 0.17	0.853

Results are presented as mean ± standard deviation. Age adjustment was performed for parametric data.

**Table 5:** Nx(+) and Nx(−), PIIINP, suPAR and homocysteine correlations with other variables.

	Nx(+)		Nx(−)	
	r	*p*	r	*p*
PIIINP with:				
suPAR	0.686	<0.001	0.814	<0.001
meanSyst	−0.357	0.035	0.017	0.905
nSyst	−0.427	0.011	−0.040	0.781
nDiast	−0.374	0.027	−0.038	0.794
nMAP	−0.422	0.014	−0.021	0.886
suPAR with:				
Natriuria	−0.458	0.006	−0.066	0.651
nDiast	−0.378	0.025	−0.072	0.620
Homocysteine with:				
BMI	−0.039	0.826	0.365	0.009
eGFR	−0.580	<0.001	−0.658	<0.001
UA	0.318	0.062	0.469	0.001
Albumin	−0.206	0.235	−0.291	0.041
Alb/Glob	−0.488	0.007	−0.417	0.003
Proteinuria	0.159	0.362	0.396	0.004
Albuminuria	0.227	0.189	0.440	0.001
meanSyst	0.209	0.229	0.310	0.028
Pulse pressure	0.256	0.138	0.414	0.003
dSyst	0.225	0.194	0.286	0.044
nSyst	0.198	0.254	0.288	0.043
CIMTr	0.101	0.564	0.355	0.011
Antihypertensives	0.275	0.110	0.325	0.021

## Data Availability

The datasets generated and analyzed in the current study are available from the corresponding author upon reasonable request.
